# Comparative Study on the effectiveness of Glycopyrrolate/Formoterol versus Tiotropium/Formoterol in patients with Chronic Obstructive Pulmonary Disease

**DOI:** 10.1016/j.conctc.2022.100931

**Published:** 2022-05-27

**Authors:** Nalini Jayanthi, Karthickeyan Krishnan, Manali Sudhir, S. Girija, Nishi P A, Sathish Kumar J

**Affiliations:** a Department of Respiratory Medicine, SRM Medical College Hospital and Research Centre, SRM Institute of Science and Technology, Kattankulathur, Tamil Nadu, 603203, India; b Department of Pharmacy Practice, SRM College of Pharmacy, SRM Institute of Science and Technology, Kattankulathur, Tamil Nadu, 603203, India; c Department of Pharmacy Practice, Vels Institute of Science and Technology and Advanced Studies, Pallavaram, Chennai, 600117, India

**Keywords:** Glycopyrrolate, Tiotropium, COPD, Formoterol, Muscarinic antagonist, Beta-_2_ agonists

## Abstract

**Background:**

Chronic Obstructive Pulmonary Disease (COPD) has several implications on health, lifestyle, and economic burden. Combinational therapy using muscarinic antagonists and beta-_2_ agonists has long been warranted for use as maintenance therapy. A lack of studies directly comparing Glycopyrrolate/Formoterol (GFF) versus Tiotropium/Formoterol (TFF) was observed which led us to analyze the effectiveness of these combinations.

**Methods:**

In this pilot, prospective, randomized, open-label, parallel-arm, 12-week period study, 60 patients with COPD (moderate-severe) were randomized in a 1:1 ratio to receive either GFF or TFF (n = 30 each). The primary outcome was to demonstrate non-inferiority between the two groups concerning FEV_1_ for 12 weeks. The secondary outcome was the assessment of the ratio of FEV_1_/FVC and state of health evaluation by St. George's Respiratory Questionnaire (SGRQ).

**Results:**

Out of 60 participants, 58 subjects completed the study. At week 12, the mean and standard deviation value of FEV_1_ between groups were 1.49 ± 0.38 and 1.38 ± 0.30 (p > 0.05) and FEV_1_/FVC ratio were 0.67 ± 0.09 and 0.74 ± 0.08 (p < 0.01) respectively. A significant difference was observed in the FEV_1_ and FEV_1_/FVC values in comparison with baseline versus last follow up in both the groups (p < 0.01). However, no remarkable variation was identified in the FEV_1_ values over the two groups. The health status assessment by SGRQ showed significant improvement in both groups after the treatment.

**Conclusion:**

Non-inferiority of GFF when compared to TFF was established along with good tolerability and comparable adverse effect profile.

## 1 Introduction

Chronic Obstructive Pulmonary Disease (COPD) is a preventable, common, and treatable disease, symbolized by incessant airflow limitation which is generally progressive and is precipitated by an increased inflammatory response in the lungs and airways due to toxic gases or particles. Symptoms such as dyspnea, sputum production, and cough have been largely reported by patients. Furthermore, the presence of underlying chronic co-morbidities contributes to its morbidity and mortality [1]. Majority of the hospital admissions related to COPD have been attributed to exacerbations (a sudden worsening of symptoms) which have enforced a compelling economic burden globally. Repeated exacerbations also lead to an overall decline in lung function, poor quality of life, and elevated risk of death [2]. According to WHO, low and middle-income countries account for 90% of COPD-related deaths. Although the primary etiological factors are considered to be cigarette smoking and air pollution, there are additional factors that increase the risk of COPD in low and middle-income countries. These include smoke from biomass fuels, other indigenous methods of smoking, previous history of tuberculosis and low socioeconomic status are the important risk factor for the development of COPD. In India, indigenous tobacco smoking methods such as hookah, chillum, bidis are more common when compared to cigarette smoking which is associated with an even greater risk [[3], [4], [5]].

Inhalational bronchodilators namely, Long Acting β-Agonists (LABAs) and Long-Acting Muscarinic Antagonists (LAMAs) prescribed either alone or as a combination with inhaled corticosteroids are the backbone for stable moderate-severe COPD management [6,7]. The rationale behind combining LABA and LAMA co-administration is an optimization of pharmacological therapy via greater bronchodilation, lesser side effects, and boost medication adherence, as only a single inhaler device is used [8].

Glycopyrronium (50 μg), a once-daily LAMA that acts on muscarinic M_1,_ M_2_, M_3_ receptors, has a high affinity for M_3_ receptors over M_2_ receptors when compared to Tiotropium. Therefore, it is hypothesized to possess lesser cardiovascular side effects than Tiotropium [9]. Glycopyrrolate/Formoterol Fumarate (GFF) is a LAMA/LABA combination drug, approved for patients with moderate-severe COPD in India in the year 2016. Tiotropium often called the gold standard of long-term COPD management, is one of the first LAMAs approved for use in COPD patients and has also shown to improve patient symptoms and health status [10]. Previous studies comparing Glycopyrrolate and Tiotropium have reported comparable efficacy and safety with Glycopyrrolate having a faster onset of action [[11], [12], [13], [14], [15]].

The current pilot study aimed to compare GFF with Tiotropium/Formoterol (TFF) combination as no direct head to head trials comparing these two combinations have been carried out and to establish the equivalence of GFF relative to TFF combination therapy.

## 2 Materials and methods

### 2.1 Selection of study subjects

Male and female participants of age ≥40 years with prior history of smoking (calculated according to pack-years) assessed with moderate-severe COPD (Stage 2 or 3 according to GOLD 2019 guidelines) were included in the study. Participants were also enrolled if they had a post-bronchodilator Forced Expiratory Volume in 1 s (FEV_1_) ≥ 30% and <80% of predicted as well as post-bronchodilator FEV_1_/Forced Vital Capacity (FVC) < 0.70 at the screening (post bronchodilator volume was assessed 1 h after inhalation of 400 μg Salbutamol and 84 μg of Ipratropium bromide). Patients who had COPD history and were already receiving treatment underwent a washout period for seven days and thereafter received their respective trial drugs according to the randomization. The condition that requires hospitalizations due to sudden worsening of symptoms defined as exacerbations were treated with antibiotics, oral corticosteroids, and bronchodilators. Rescue medication use was also recorded. The trial drugs were then continued when an improvement in the symptoms after the management of exacerbation was observed.

Participants with the following conditions were excluded from the study: i) Pregnant and/or nursing mothers (ii) Women of childbearing potential-unless adequate contraceptive measures were being taken (iii) Known underlying cardiovascular abnormalities (arrhythmias, congestive heart failure, coronary artery disease) (iv) Renal impairment (v) Patients with urinary retention (vi) Narrow-angle glaucoma (vii) familiar history of psychiatric illness (viii) Undergone surgeries including lobectomy or bronchoscopic lung volume reduction (ix) Inability to produce acceptable spirometry results (x) Contraindications/hypersensitivity to any of the study drugs (xi) History of adverse reactions to inhaled anticholinergics (xii) Known malignancies.

## 2.2 Ethical approval

This study has approval from Institutional Ethics Committee (1785/IEC/2019) of SRM Medical College Hospital and Research Centre and the research was performed in abidance to Declaration of Helsinki. The patients were subjected to treatment allocation only after obtaining the written informed consent for participation in the study. This research has also been registered under the Clinical Trial Registry of India (Registration number CTRI/2020/01/022780).

### 2.3 Study design

A randomized, prospective, pilot, open-label, parallel-arm study was undertaken in a tertiary care hospital in Chengalpattu district, Tamil Nadu for 12 weeks. A sample size of approximately 30 in each group was determined at 80% power of the study and an alpha value of 0.05 for a two-arm parallel study. The maximum clinically significant allowable difference of FEV_1_ between two groups used 50 mL (0.05L) and pooled standard deviation 200 mL (0.2L) were extracted from the study by Chapman et al. (GLOW-5) [11]. Block randomization method was followed using a block size of 4 and randomization sequencing was created using Random Allocation Software (version 2.0). Allocation concealment was done using Sequentially Numbered, Opaque Sealed Envelopes (SNOSE). One investigator who did not participate in patient recruitment prepared the sealed envelopes. Spirometry was performed in order to assess the study outcomes.

#### 2.3.1 Intervention

A total of 60 subjects were assigned randomly in a ratio of 1:1 to either one of the two groups (Group A or Group B). Subjects in Group A received GFF Dry Powder Inhaler (DPI) (25 μg/6 μg) twice daily whereas participants in Group B received TFF DPI (18μg/12 μg) once daily.

### 2.4 Study outcomes and assessment

The primary outcome of the study was to demonstrate non-inferiority between the two groups in terms of improvement in the FEV_1_ values by the end of week 12. The secondary outcome was an improvement in FEV_1_/FVC ratio and health status. FEV_1_/FVC ratio and FEV_1_ values were assessed using spirometry at baseline, weeks 4, 8, and 12 (EasyOne spirometer, NDD Medical Technologies). Health status was evaluated using St. George's Respiratory Questionnaire (SGRQ) [16]. Eligibility was assessed by checking for post-bronchodilator reversibility upon inhalation of Salbutamol (2.5 mg). Side effects reported by the patients at every follow-up were recorded and assessed using the Naranjo scale.

### 2.5 Statistical analysis

The demographic and baseline characteristics were represented by descriptive statistics. The normality distribution of the data was analyzed using the Kolmogorov Smirnov test. The primary and secondary outcomes were analyzed using repeated measures Analysis of Variance (ANOVA) and post hoc Bonferroni tests for pairwise comparisons using Statistical Product and Service Solutions (SPSS) Version 27.

## 3 Results

### 3.1 Demographic and baseline characteristics

**Fig. 1:**
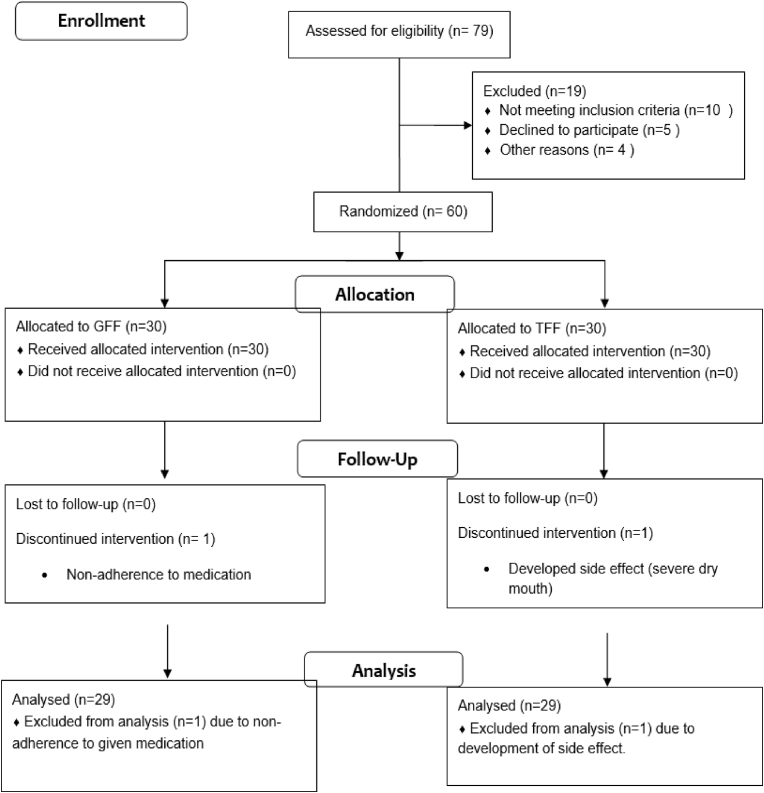
CONSORT Flow diagram of study population.

A total number of 79 patients were selected and screened for eligibility, of which 60 patients were randomized into two groups. 30 subjects were allocated in the GFF group and 30 subjects were in the TFF group. Completion of the study was achieved by 58 patients. The dropout rate was similar in both groups with the prime reasons being non-adherence to the medication and withdrawal due to adverse drug reaction (Fig. 1).

The disposition of the baseline characteristics was tabulated (Table 1). The mean age distribution between the groups was 52.5 and 69.5 years respectively. The majority of the patients over the groups had moderate to severe COPD. The mean duration of COPD was found to be 4.6 years. In both the groups, over half of the patients had a positive smoking history. The mean post-bronchodilator FEV_1_ predicted percentage was 61% and the post-bronchodilator FEV_1_/FVC ratio was 64%. The percentage of diabetic patients was highest in each of the two groups followed by hypertensive patients.

**Table 1:** Baseline demographic details of patients across groups. FEV1: Forced expiratory volume in 1 s; FVC: Forced vital capacity; SD: Standard deviation; Group A – Glycopyrrolate/formoterol; Group B- Tiotropium/formoterol.

PARAMETERS	GROUP A N = 29	GROUP B N = 29
**Age in years [mean (SD)]**	52.93(7.00)	69.53(4.07)
**Gender n (%)**		
Male n (%)	21(72)	18(62)
Female n (%)	8(28)	11(38)
BMI, mean(SD)	23.17(2.67)	23.0 (4)
**Severity of disease n (%)**		
Moderate	17(59)	21(72)
Severe	11(38)	7(24)
Very severe	1(3)	1(3)
**Smoking status n (%)**		
Non-smokers	16(55)	13(45)
Former smokers	8(28)	11(38)
Current smokers	5(17)	5(17)
**Duration of smoking, Pack years mean(SD)**	30.69(25.87)	24.44(22.8)
**Duration of COPD years, mean(SD)**	4.67 (5.6)	4.73(6.1)
**FEV**_**1**_**post-bronchodilator (L) mean(SD)**	1.36(0.40)	1.18(0.35)
**FEV**_**1**_**post-bronchodilator % predicted, mean (SD)**	60(12)	62(19)
**FEV**_**1**_**/FVC post-bronchodilator % Mean (SD)**	63(9)	65(8)
**Comorbidities N (%)**		
Hypertension	3 (10%)	6 (20%)
Diabetes	6 (20%)	6 (20%)
Past TB	3 (10%)	3 (10%)
Others	3 (10%)	2 (7%)

### 3.2 Spirometry observations

The primary outcome was an improvement in FEV_1_ values (in liters). We performed two way repeated measures ANOVA to check if there was any significant difference in the average FEV_1_ at different time points as well as between groups. It was observed that there was a significant difference in the average FEV_1_ from baseline up to the 12th week of study (p < 0.01). Post-hoc Bonferroni pairwise comparison of the means revealed that there was significant progress in the average FEV_1_ values among all the pairs of time points (p < 0.01) (Table 2). Furthermore, the results of FEV_1_ between both the treatment groups shows no remarkable variation (p = 0.14).

**Table 2:** Mean and SD changes of FEV_1_ across treatment groups. FEV_1_- Forced expiratory volume in second; SD – Standard deviation. Group A – Glycopyrrolate/formoterol; Group B- Tiotropium/formoterol.

Groups	FEV_1_ [Mean (SD)]				F value	P-value
	Baseline	4th week	8th week	12th week		
Group A [n = 29]	1.24 (0.38)	1.39 (0.44)	1.45 (0.37)	1.49 (0.38)	117.34	<0.01
Group B [n = 29]	1.08 (0.32)	1.23 (0.33)	1.33 (0.31)	1.38 (0.30)		

The secondary outcome was to apprehend important difference in the average FEV_1_/FVC at various time points, between treatment groups. A statistically significant difference was found in the pattern of average FEV_1_/FVC over the time between groups GFF and TFF (p = 0.02). The post-hoc comparison have shown a significant difference in the average FEV_1_/FVC (p < 0.01) values between all pairs of time points (Table 3). 5 patients in the GFF group and 4 patients in the TFF group reported using rescue medication during the study period for exacerbations.

**Table 3:** Mean and SD changes of FEV_1_/FVC ratio across groups. FEV_1_- Forced Expiratory Volume in 1 s; FVC-Forced Vital Capacity; SD- Standard Deviation; Group A – Glycopyrrolate/formoterol; Group B- Tiotropium/formoterol.

	FEV_1_/FVC Mean (SD)				F value	P-value
	Baseline	4th week	8th week	12th week		
Group A [n = 29]	0.60 (0.09)	0.64 (0.09)	0.66 (0.09)	0.67 (0.09)	67.49	<0.01
Group B [n = 29]	0.63 (0.08)	0.68 (0.09)	0.73 (0.09)	0.75 (0.08)		

The health status, quality of life assessment were analyzed using SGRQ which consists of three domains-symptom, activity, and impact scores which are added to give a total score. There was a considerable difference in the average total score including symptom, impact, and activity before and after the intervention (p < 0.01). The results from the post-hoc analysis show no significant difference in the score between group A and group B (p = 0.61) (Table 4).

**Table 4:** Mean differences of SGRQ scores between treatment groups before and after an intervention. SGRQ-St. George respiratory questionnaire; SD- Standard deviation.

Groups	SGRQ Total score		F value	P-value
	Before intervention Mean (SD)	After intervention Mean (SD)		
Group A [n = 29]	54.14 (16.07)	31.01 (12.67)	191.35	<0.01
Group B [n = 29]	52.83 (13.45)	30.65 (11.69)		

### 3.3 Adverse drug reactions

The incidence of adverse drug reactions was more in number in the TFF group (Group B) in comparison to GFF (Group A). The most commonly reported adverse effects in Group B were dry mouth, constipation, and frequent urination/urinary retention. Increased secretions and dyspnea were periodically encountered in the GFF group. One patient withdrew from the study due to severe dry mouth in the TFF group (Table 5).

**Table 5:** Adverse drug reactions in treatment groups.

Adverse reactions reported	Group A N = 29 N (%)	Group B N = 29
Dry mouth	3(10)	9(31)
Cough	6(20)	7(23)
Constipation	1(3)	3(10)
Increased secretions	4(13)	1(3)
Blurred vision	0	1(3)
Dyspnea	7(23)	2(6)
Frequent urination/urinary retention	2(7)	5(17)

## 4 Discussion

Our pilot study showed a significant improvement in our set primary and secondary spirometry endpoints. We found that GFF was non-inferior to TFF in improving COPD patient outcomes. A post-hoc analysis was done to better comprehend differences or improvements from baseline to the last time point (12th week). Two landmark studies, namely the GLOW2 and GLOW5 [11,14] clinical trials directly compared the safety and efficacy of Glycopyrronium and Tiotropium in head-to-head trials, and their results were found similar to the current study. A significant improvement in FEV_1_/FVC ratio at baseline to 12th week was found in both these studies and was comparable to the prevailing study [11,12]. Patients with COPD are known to have severe morning symptoms which have repercussions on their daily tasks. The utilization of LAMAs is postulated to play a vital role in the improvement of these symptoms. Our results were also analogous to the SPRING [17] study wherein glycopyrrolate was compared to blinded tiotropium to improve these morning symptoms and non-inferiority of the former drug was established.

Our study also showed a similar progression from initiation of treatment when the status of health of patients was assessed from the SGRQ. Larger clinical trials have also reported similar results [11,17]. Studies have also reported glycopyrrolate to have a faster onset of action when compared to tiotropium but this was not evident on repeated administration of dose [18,19].

Currently, it is not precisely acquainted if there had been any differences in safety and efficacies amongst other LAMA/LABAs. More clinical trials are imperative to confirm this as the COPD burden is on a rising trend and more patients would potentially require more than one LABA or LAMA for better management of their symptoms.

The safety of these drugs was also comparable as the frequency of adverse drug reactions was similar. The Glycopyrrolate/Formoterol combination shows a persistent safety profile in comparison with its components, placebo and open-label tiotropium in its phase 3 clinical development studies (PINNACLE-1, 2; PT003011and PT003012) [[20], [21], [22]].

We were unable to find relevant studies comparing the same combination as our study drugs. Limitations of this study were chiefly the small sample size due to it being a pilot study and the short time duration of the study was also a factor. Thirdly, the trial was an open-label study which could have led to selection bias.

## 5 Conclusion

The results of the present study indicated that the Glycopyrrolate/Formoterol combination was non-inferior to TFF with a similar safety profile and good tolerability. On comparing the safety profiles, GFF had better tolerability to TFF and can be used in those patients who cannot tolerate the adverse effects posed by TFF. Therefore, GFF can be considered for use instead of TFF in the long-term maintenance of COPD.

## Funding

This research did not receive any specific grant from funding agencies in the public, commercial, or not-for-profit sectors.

## Author's contribution

**Nalini Jayanthi**: Conceptualization, Methodology, Supervision, Project Administration, Validation; **Karthikeyan**: Supervision, Project Administration; **Manali Sudhir**, Investigations, Writing-Original Draft Preparation; **Girija:** statistical analysis and interpretation, Writing-Review & Editing, Formal Analysis, **Sathish Kumar**: Investigation; **Nishi**: Investigation.

## Declaration of interests

The authors declare that they have no known competing financial interests or personal relationships that could have appeared to influence the work reported in this paper.

## Abbreviations

COPD: Chronic Obstructive Pulmonary Disease
DPI: Dry Powder Inhaler
FEV_1_: Forced Expiratory Volume in 1 s
FVC: Forced Vital Capacity
GFF: Glycopyrrolate/Formoterol
GOLD: Global Initiative for Chronic Obstructive Lung Disease
ICS: Inhaled Corticosteroid
LABA: Long-Acting Beta Agonist
LAMA: Long-Acting Muscarinic Antagonist
SGRQ: St. George's Respiratory Questionnaire
SNOSE: Serially Numbered Opaque Sealed Envelopes
TFF: Tiotropium/Formoterol

## References

[1] Global initiative for Chronic Obstructive Lung Disease Global strategy for the diagnosis, management, and prevention of Chronic Obstructive pulmonary disease 2019 report. https://goldcopd.org/wp-content/uploads/2018/11/GOLD-2019-v1.7-FINAL-14N.

[2] Qureshi H., Sharafkhaneh A., Hanania N.A. (2014). Chronic obstructive pulmonary disease exacerbations: latest evidence and clinical implications. Ther. Adv. Chronic Dis..

[3] Rajkumar P., Pattabi K., Vadivoo S., Bhome A., Brashier B., Bhattacharya P., Mehendale S.M. (2017). A cross-sectional study on prevalence of chronic obstructive pulmonary disease (COPD) in India: rationale and methods. BMJ Open.

[4] Rhee C.K., Yoshisue H., Lad R. (2019). Fixed-dose combinations of long-acting bronchodilators for the management of COPD: global and asian perspectives. Adv. Ther..

[5] Gupta D., Agarwal R., Aggarwal A.N., Maturu V.N., Dhooria S., Prasad K.T., Sehgal I.S., Yenge L.B., Jindal A., Singh N., Ghoshal A.G., Khilnani G.C., Samaria J.K., Gaur S.N., Behera D., S. K. Jindal for the COPD Guidelines Working Group (2013). Guidelines for diagnosis and management of chronic obstructive pulmonary disease: joint ICS/NCCP (I) recommendations. Lung India: Off. Organ Indian Chest Soc..

[6] Wang M.T., Lai J.H., Tsai C.L., Liou J.T. (2019). Risk of adverse cardiovascular events with use of inhaled long-acting bronchodilators in management of chronic obstructive pulmonary disease. J. Food Drug Anal..

[7] Weiss A., Porter S., Rozenberg D., O'Connor E., Lee T., Balter M., Wentlandt K. (2020). Chronic obstructive pulmonary disease: a palliative medicine review of the disease, its therapies, and drug interactions. J. Pain Symptom Manag..

[8] Radovanovic Dejan, Mantero Marco, Valenti Vincenzo, Aliberti Stefano, Di Marco Fabiano, Santus Pierachille, Giuseppe Francesco Sferrazza Papa (2016). Formoterol fumarate + glycopyrrolate for the treatment of chronic obstructive pulmonary disease. Expet Rev. Respir. Med..

[9] Buhl R., Banerji D. (2012). Profile of glycopyrronium for once-daily treatment of moderate-to-severe COPD. Int. J. Chronic Obstr. Pulm. Dis..

[10] Tashkin D.P., Celli B., Senn S., Burkhart D., Kesten S., Menjoge S., Decramer M., UPLIFT Study Investigators (2008). A 4-year trial of tiotropium in chronic obstructive pulmonary disease. N. Engl. J. Med..

[11] Chapman K.R., Beeh K.M., Beier J., Bateman E.D., D'Urzo A., Nutbrown R., Henley M., Chen H., Overend T., D'Andrea P. (2014). A blinded evaluation of the efficacy and safety of glycopyrronium, a once-daily long-acting muscarinic antagonist, versus tiotropium, in patients with COPD: the GLOW5 study. BMC Pulm. Med..

[12] Wang C., Sun T., Huang Y., Humphries M., Bai L., Li L., Wang Q., Kho P., Firth R., D'Andrea P. (2015). Efficacy and safety of once-daily glycopyrronium in predominantly Chinese patients with moderate-to-severe chronic obstructive pulmonary disease: the GLOW7 study. Int. J. Chronic Obstr. Pulm. Dis..

[13] Beeh K.M., Singh D., Di Scala L., Drollmann A. (2012). Once-daily NVA237 improves exercise tolerance from the first dose in patients with COPD: the GLOW3 trial. Int. J. Chronic Obstr. Pulm. Dis..

[14] Kerwin E., Hébert J., Gallagher N., Martin C., Overend T., Alagappan V.K., Lu Y., Banerji D. (2012). Efficacy and safety of NVA237 versus placebo and tiotropium in patients with COPD: the GLOW2 study. Eur. Respir. J..

[15] D'Urzo A., Ferguson G.T., van Noord J.A., Hirata K., Martin C., Horton R., Lu Y., Banerji D., Overend T. (2011). Efficacy and safety of once-daily NVA237 in patients with moderate-to-severe COPD: the GLOW1 trial. Respir. Res..

[16] Jones P.W. (2005). St. George's respiratory questionnaire: MCID. COPD.

[17] Marin J.M., Beeh K.M., Clemens A., Castellani W., Schaper L., Saralaya D., Gunstone A., Casamor R., Kostikas K., Aalamian-Mattheis M. (2016). Early bronchodilator action of glycopyrronium versus tiotropium in moderate-to-severe COPD patients: a cross-over blinded randomized study (Symptoms and Pulmonary Function in the moRnING). Int. J. Chronic Obstr. Pulm. Dis..

[18] Watz H., Mailänder C., May C., Baier M., Kirsten A.M. (2017). Fast onset of action of glycopyrronium compared with tiotropium in patients with moderate to severe COPD - a randomized, multicentre, crossover trial. Pulm. Pharmacol. Therapeut..

[19] Rennard S., Fogarty C., Reisner C. (2014). Randomized study of the safety, pharmacokinetics, and bronchodilatory efficacy of a proprietary glycopyrronium metered-dose inhaler in study patients with chronic obstructive pulmonary disease. BMC Pulm. Med..

[20] Martinez F.J., Rabe K.F., Ferguson G.T., Fabbri L.M., Rennard S., Feldman G.J., Sethi S., Spangenthal S., Gottschlich G.M., Rodriguez-Roisin R., Arora S., Siler T.M., Siddiqui S., Darken P., Fischer T., Maes A., Golden M., Orevillo C., Reisner C. (2017). Efficacy and safety of glycopyrrolate/formoterol metered dose inhaler formulated using Co-suspension delivery technology in patients with COPD. Chest.

[21] Hanania N.A., Tashkin D.P., Kerwin E.M., Donohue J.F., Denenberg M., O'Donnell D.E., Quinn D., Siddiqui S., Orevillo C., Maes A., Reisner C. (2017). Long-term safety and efficacy of glycopyrrolate/formoterol metered-dose inhaler using novel Co-Suspension™ Delivery Technology in patients with chronic obstructive pulmonary disease. Respir. Med..

[22] Reisner C., Gottschlich G., Fakih F., Koser A., Krainson J., Delacruz L., Arora S., Feldman G., Pudi K., Siddiqui S., Orevillo C., Maes A., St Rose E., Martin U. (2017). 24-h bronchodilation and inspiratory capacity improvements with glycopyrrolate/formoterol fumarate via co-suspension delivery technology in COPD. Respir. Res..

